# Mobile-Based Oral Chemotherapy Adherence–Enhancing Interventions: Scoping Review

**DOI:** 10.2196/11724

**Published:** 2018-12-21

**Authors:** Xiomara Skrabal Ross, Kate M Gunn, Pandora Patterson, Ian Olver

**Affiliations:** 1 Cancer Research Institute University of South Australia Adelaide Australia; 2 School of Health Sciences University of South Australia Adelaide Australia; 3 Cancer Nursing Research Unit University of Sydney Sydney Australia; 4 Research, Evaluation, and Social Policy Team CanTeen Australia Sydney Australia

**Keywords:** medication adherence, antineoplastic agents, neoplasms, cell phone, text messaging, mobile apps, review, mHealth

## Abstract

**Background:**

Adherence to oral chemotherapy is crucial to maximize treatment outcomes and avoid health complications in cancer patients. Mobile phones are widely available worldwide, and evidence that this technology can be successfully employed to increase medication adherence for the treatment of other chronic diseases (eg, diabetes) is well established. However, the extent to which there is evidence that mobile phone–based interventions improve adherence to oral chemotherapy is unknown.

**Objective:**

This scoping review aims to explore what is known about mobile phone–delivered interventions designed to enhance adherence to oral chemotherapy, to examine the reported findings on the utility of these interventions in increasing oral chemotherapy adherence, and to identify opportunities for development of future interventions.

**Methods:**

This study followed Arksey and O’Malley’s scoping review methodological framework.

**Results:**

The review search yielded 5 studies reporting on 4 interventions with adults (aged >18 years) diagnosed with diverse cancer types. All interventions were considered acceptable, useful, and feasible. The following themes were evident: text messages and mobile apps were the main methods of delivering these interventions, the 2 most commonly employed oral chemotherapy adherence–enhancing strategies were management and reporting of drug-related symptoms and reminders to take medication, the importance of stakeholders’ engagement in intervention design, and the overall positive perceptions of delivery features. Areas for future research identified by this review include the need for further studies to evaluate the impact of mobile phone–delivered interventions on adherence to oral chemotherapy as well as the relevance for future studies to incorporate design frameworks and economic evaluations and to explore the moderator effect of high anxiety, poor baseline adherence, and longer time taking prescribed drug on adherence to oral chemotherapy.

**Conclusions:**

Despite the increasing body of evidence on the use of mobile phones to deliver medication adherence–enhancing interventions in chronic diseases, literature on the oral chemotherapy context is lacking. This review showed that existing interventions are highly acceptable and useful to cancer patients. The engagement of stakeholders as well as the use of a design framework are important elements in the development of mobile phone–delivered interventions that can be translated into oncology settings.

## Introduction

### Background

The widespread increase in the use of chemotherapy delivered via the oral route is transforming oncology. However, self-administration of oral chemotherapy encompasses a number of challenges for patients and health professionals to ensure adequate management of adherence and toxicities [1]. Nonadherence can reduce treatment efficacy and lead to dangerous health complications, including death [2]. The rates of adherence to oral antineoplastic agents can be as low as 46% [3]. Despite this fact, most health institutions do not practice standardized patient monitoring procedures for adherence [4].

Adherence is defined as the extent to which a persons’ medication-intake behavior corresponds with the agreed recommendations of the clinician [5]. Adequate oral medication adherence is also important for the optimal treatment of other chronic conditions (eg, diabetes and HIV). Due to the long-term nature of these diseases, adherence and monitoring are required over long periods, which can be problematic. Technology is increasingly being used to help chronically ill patients adhere to their treatment regimens [6]. Mobile phones are a technological platform that allows delivery of behavioral interventions, assessments, and real-time data collection [7] and, importantly, can also facilitate access to support patients who, due to their remote geographical location or limited mobility, cannot access face-to-face services. Mobile text messages (short message service, SMS) and mobile apps are 2 types of mobile phone–based technology that are most commonly used to support patients with chronic diseases [6].

Worldwide availability of mobile phones is extensive and ownership of these technologies will continue to grow. As a result, there is great potential to use mobile technology to improve health care delivery. In 2016, 62.9% of the world population (4.65/7.40 billion) owned a mobile phone and this figure is set to increase to 67% (5.16/7.71 billion) in 2019 [8]. The introduction of smartphones means that mobile phones are no longer limited to being a tool for calls and text messages but also allow internet connectivity. Mobile devices (including smartphones and tablets) are currently the main source of internet connection. In 2017, approximately three-fourths of the worldwide internet access occurred through mobile devices [9]. In Australia, in 2016, 84% of the population (approximately 16 million people) owned a smartphone. The only places in the world where uptake of smartphones is greater are South Korea, the Netherlands, and Norway [10].

Previous research has already established that interventions delivered via mobile phones can significantly improve medication adherence for people with arterial hypertension [11], heart failure [12], and diabetes [13,14]. Moreover, acceptability and usefulness of mobile phone–delivered interventions are known to be high among chronically ill patients [6]. However, it is important to note that the efficacy of adherence-enhancing interventions is determined by the quality of the strategies delivered by a form of technology. Gains should not be simply attributed to the type of technology employed.

Although the reach, popularity, and many technological features of mobile phones now mean they are ideal platforms to provide health care support to cancer patients undergoing oral chemotherapy, because the widespread use of oral chemotherapy drugs is relatively new, the extent to which evidence is available to support this strategy is unknown.

### Objectives

To address the emerging issue of oral chemotherapy nonadherence, this scoping review aims to:

- explore what is known about oral mobile phone–delivered interventions designed to enhance adherence to oral chemotherapy,
- examine the reported findings on the utility of mobile phone–delivered interventions in increasing adherence to oral chemotherapy, and
- identify opportunities for future development of oral chemotherapy adherence–enhancing interventions via mobile phone.

## Methods

### Overview of Methods

The scoping review methodological framework used in this review was outlined by Arksey and O’Malley [15]. This approach is ideal to understand research fields that are in early stages because it allows the rapid mapping of key concepts, sources, and evidence available, leading to identification of gaps in the existing literature [15]. This method aims to produce broad results from all relevant literature instead of trying to answer highly focused questions from specific study designs, as is the case in systematic reviews. Consistent with Arksey and O’Malley’s framework [15], this study presents a narrative review of literature based on an analytic framework (thematic analysis) [16] and does not seek to assess the quality of studies, including risk of bias or generalizability of findings.

### Identification of Relevant Studies

A structured database search was conducted in April 2018 with the following databases: MEDLINE, EMBASE, EMcare, and PsycINFO using terms related to 5 key areas: mobile phones, adherence, intervention, oral chemotherapy, endocrine therapy, and cancer. Subject headings and keywords used in databases and independent reviews were collated for each of the 5 key areas using the “OR” function and groups 1 to 5 were connected with the “AND” function. Examples of keywords included in each area were (1) mobile phone, text messaging, mHealth, mobile app; (2) patient compliance, medication compliance, medication adherence; (3) program, pilot, study, review, randomized controlled trial; (4) oral chemotherapy, antineoplastic agents, oral anti-cancer, endocrine therapy; and (5) neoplasm, tumour, cancer. Subject headings (eg, Medical Subject Headings) were employed. English language limits were applied, but no date restrictions were used for this search. Grey literature was searched through the ProQuest Dissertations and Theses database, limited to doctoral theses between January 2013 and March 2018 due to the high number of irrelevant results obtained with unlimited searching. To extend the results, an independent search in a Web journal took place. Reference lists of relevant articles were also reviewed for references that may have been missed when conducting the database research.

Conference proceedings were included to make this review as broad and informative as possible. Titles and abstracts of retrieved documents were screened against inclusion criteria, followed by full text review of relevant studies.

### Selection of Studies

To include relevant studies and associated content that contributed to meeting the objectives of this review, the following inclusion criteria were used to guide the screening process: (1) research-based studies on interventions that aim to increase adherence to oral chemotherapy or endocrine therapy, (2) targets cancer patients taking oral chemotherapy or endocrine therapy, (3) use of mobile phones as a main tool to deliver the intervention, and (4) articles written in English. In this study, adherence was defined as taking oral chemotherapy in accordance with the dose and frequency prescribed by the clinician.

### Data Charting

Full text articles were assessed to extract relevant information, and this was transferred into an Excel spreadsheet. Information charted in this process was as follows: (1) authors, (2) study purpose, (3) research design (eg, qualitative and randomized controlled trial [RCT]), (4) participants (age, cancer diagnosis, oral chemotherapy or endocrine treatment, and country), (5) mobile phone features (eg, text messages and apps), (6) intervention (duration and key components of intervention), and (7) main findings (summary of the most relevant findings, including recommendations for future studies). A second reviewer assessed 50% (5/10) of articles to ensure validity of information extraction.

### Thematic Analysis and Reporting of Results

Following the methodological framework from Arksey and O’Malley [15], thematic analysis [16] was used to identify what is already known about mobile phone–based oral chemotherapy adherence–enhancing interventions, the utility of interventions in improving oral chemotherapy adherence, and the opportunities for future development in this area. A second reviewer assessed all codes and a complete agreement was achieved. Due to the research designs used in the reviewed studies (most were nonexperimental), results are reported in category groups conformed by common themes in the reviewed literature. Categories are aligned with the aims of this research.

## Results

### Overview of Results

**Figure 1:**
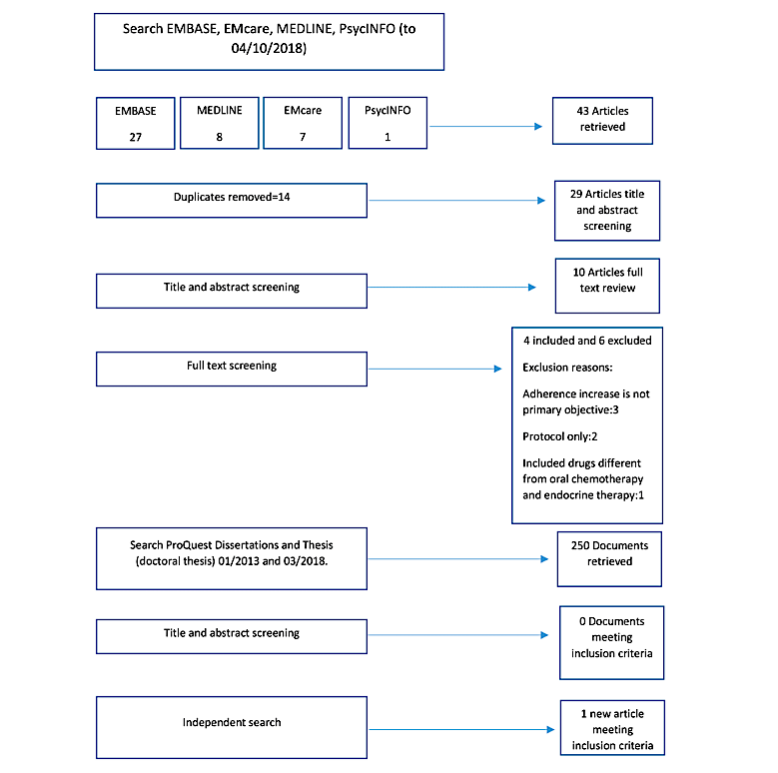
Flowchart of study selection process and included studies.

During the initial database search, 43 articles were retrieved. After removal of duplications, 29 unique publications were identified. Titles and abstracts were scanned for relevance against the inclusion criteria and those that did not match (eg, focus on another illness or lack of focus on improving oral chemotherapy adherence) were removed, leaving 10 articles for full text review. After full text review, 6 articles were excluded. An additional publication was added following an independent review in an online journal. No articles fitting the inclusion criteria were identified through the grey literature search in the ProQuest Dissertations and Theses database or through search in articles’ reference lists (Figure 1 shows the detailed process of screening and inclusion). Ultimately, 5 articles [17-21] reporting 4 interventions were included in this review, 2 of which were abstracts from conference proceedings and 3 were peer-reviewed journal articles. Moreover, 1 study [19] describes the development of an intervention that was later tested in another one of the included articles [18]. Multimedia Appendix 1 shows the information extracted.

Search results showed that research on mobile phone–based interventions to increase adherence to oral chemotherapy began in 2015. The research designs of the studies were qualitative (3 out of 5 studies) [19-21] and experimental RCTs (2 out of 5 studies) [17,18]. All but 1 study explored the feasibility and acceptability of interventions, and 2 out of 5 studies used an RCT design to evaluate the effect of mobile phone interventions on adherence to oral chemotherapy [17,18].

Moreover, 3 out of the 5 studies had small samples (5-32 participants) [19-21]; the remaining 2 studies used an RCT design with larger samples (80 and 181 participants) to measure oral chemotherapy adherence as a primary outcome [17,18].

Of the 5 studies, 3 included participants with diverse types of cancer [17-19], 1 focused on patients with chronic myeloid leukemia [20], and 1 focused on breast cancer patients [21]. All the studies focused on adult participants (aged >18 years).

### Strategies and Features of Intervention Delivery

Various methods of delivery and adherence-enhancing strategies were employed in the reviewed studies. In addition, 2 distinct methods were used to deliver the interventions: SMS text messages and mobile apps. The SMSs were sent as medication intake reminders and frequency was daily or twice daily according to individual medication intake schedules [17,20]. The SMS reminders were bidirectional (participants’ response “Y” or “N” was expected) to collect data on the frequency of taking medication. The content of the SMS (message bank, wording, and theoretical framework) was reported by Spoelstra et al [17] and informed by social cognitive theory. The SMS content in Pereira-Salgado et al’s study was not described [20]. For future research to understand the process of how SMS reminders influence adherence, it is important that these details (eg, content, frequency, and sender) are reported. Overall, participants showed high rates of response to SMS reminders. In 2 studies, they replied 87.13% (1036/1189) of the times [17] and only up to 22% (17/80) of the SMS were not replied when sent [20]. This not only shows that participants received and read the SMS but also actively participated in reporting medication-taking events.

The SMS reminders in the study by Pereira-Salgado et al were part of an intervention that also included a Web-based app. It recorded participants’ side effects and severity and provided real-time self-management advice [20]. This intervention also incorporated nurse-led phone support consultations. This last strategy was not necessarily delivered via mobile phone.

Mobile apps were used as delivery features in 2 interventions [18,21]. They all included reminders to take medication. Daily reminders (alarms) were set by users according to their preferences in 1 study [19]. Brett et al’s study did not report on any characteristics of medication reminders (eg, frequency and type) [21].

Other strategies used in mobile app interventions were real-time side effect management advice, cancer-specific information, medication-specific information, and chat forums (Table 1 shows features and strategies of each of the interventions). Automatic generation of reports accessible by research teams was another technical element in 1 mobile app and a combined Web app and SMS intervention. Reports contained data on symptom severity [19] or a combination of medication-taking and symptom severity [20].

The potential influence of oral chemotherapy-induced side effects on adherence to oral chemotherapy was recognized widely in the reviewed studies. Strategies to improve symptom management were incorporated in three-fourths of the interventions. These strategies included symptom reports with real-time reception of tailored feedback [18,21] and symptom recording in a side effect mobile app diary [21]. Symptom burden and severity was also 1 of the primary outcomes in most interventions (3 out of 4 studies) [17,18,20].

**Table 1:** Strategies and features of interventions.

Strategy or feature		Spoelstra et al, 2016 [17]	Greer et al, 2017 [18]	Pereira-Salgado et al, 2017 [20]	Brett et al, 2018 [21]
**Strategy**					
	Medication reminders	✓	✓	✓	✓
	Symptom management information	—^a^	✓	✓	✓
	Cancer-specific information	—	✓	—	✓
	Individual nurse phone support	—	—	✓	—
	Medication-specific information	—	✓	—	✓
	Recording of side effects	—	✓	✓	✓
	Chat forum	—	—	—	✓
**Feature**					
	SMS^b^ text messages	✓	—	✓	—
	Mobile app	—	✓	—	✓
	Web-based app	—	—	✓	—

^a^Strategy or feature not present.

^b^SMS: short message service.

### Acceptability, Usability, and Feasibility of Interventions

Overall, all interventions reviewed were found to be acceptable and useful, and their implementation was feasible. The definition of acceptability differed across studies. Spoelstra et al’s study defined acceptability as the percentage of patients who agreed to take part in the study and the percentage of patients who completed the study [17]. This SMS medication reminder intervention had high rates of acceptability among cancer patients, with 75.7% (78/103) of eligible potential participants consenting to participate and 86% (42/49) of the initial participants completing the entire intervention.

Preliminary acceptability of ChemOtheRapy Assistant (CORA) mobile app [19] was described as the result of a 5-stage developmental process that led to intervention improvement. Stakeholders (eg, patients, and oncology clinicians) participated in qualitative research to provide information that led to the design of an acceptable final version of the mobile app.

Pereira-Salgado et al’s REMIND system study assessed intervention acceptability by interviewing participants (patients and nurses) after using the system [20]. The intervention was highly acceptable to participants in terms of its content, timing, and perceived utility of each component (SMS medication reminders, symptom management advice, and nurse phone support). Usability was assessed separately in this study with participants expressing appreciation for the ease of use of text reminders and the weekly symptoms component, with the exception of a small number of participants who expressed delays in receipt of the SMS. The nurses also found the intervention useful but suggested changes to the number of report emails and layout of the intervention manual (ie, inclusion of tabs to facilitate the search for specific content).

The definitions of acceptability and usefulness in Brett et al’s study were not provided [21]. This conference proceeding indicated that participants considered individual components of the mobile app intervention (information section, links to evidence around adjuvant endocrine therapy, side effects diary, and repeat prescription reminders) acceptable and useful. However, perceptions of the usefulness of the chat forum were mixed. Participants also suggested more information on side effect management strategies.

Measures of feasibility also varied across studies. Measures included the number of SMS delivered and returned [17] and possibility to implement and integrate the intervention into a clinical setting [20]. Brett et al’s study, which aimed to explore the feasibility of a mobile app intervention, reported results in terms of high acceptability, usefulness, and usability of the intervention [21]. The CORA exploratory study [19] did not report on the intervention feasibility. However, its later implementation in clinical settings with 181 oral chemotherapy users can be considered confirmation of feasibility [18].

### Stakeholders’ Engagement in the Design of Interventions

Stakeholders’ engagement was evidenced in the design of all the reviewed interventions, through the exploration of end users’ perceptions of the acceptability and usefulness of mobile phone interventions. An example of this is the design of the CORA mobile app, which was informed at every stage by groups of stakeholders: patients and caregivers; oncology clinicians; cancer practice administrators; and representatives of the care system, community, and society [19]. Engagement of these groups allowed the intervention to be shaped by users’ opinions on the need for oral chemotherapy self-management support; the role of the intervention in supporting oral chemotherapy self-management, acceptability, and usability; as well as exploring possible implementation barriers [19].

Cancer patients also participated in early design phases of Pereira-Salgado et al’s REMIND system for patients with chronic myeloid leukemia [20] and Brett et al’s mobile app for women taking adjuvant endocrine therapy after breast cancer [21]. The first study also included oncology clinicians and explored their perceptions of the nature, extent, and reasons for nonadherence to tyrosine kinase inhibitors. The second study examined patients’ preferences on the content of a mobile app [21]. Findings from both studies informed the strategies incorporated into mobile phone interventions, in line with patients’ needs and preferences.

Spoelstra et al’s study [17] assessed, among other variables, participants’ acceptability and satisfaction with the intervention, and both were rated as high by study participants. These findings support the possibility of cancer patients to incorporate this text message intervention into their daily lives.

### Design Framework Informing Development of Mobile Phone Interventions

Overall, 2 of the 4 interventions [19,20] reported the use of a design framework as a guide during the development process. In the design of the CORA mobile app [19], the investigators incorporated Whittaker et al’s framework [22], which sets a process that involves steps to develop and test mobile phone–based health interventions. In doing this, the investigators based the design on a theoretical model, conducted formative research, pretested the intervention with stakeholders, and piloted the app with 5 participants enrolled in the next phase of the research, which was an RCT. Results from the experimental phase were reported in a separate study [18], and qualitative follow-up was also intended to be measured in the same study. However, due to the nature of the article (conference proceeding), detailed information on this was not provided.

Fishbein et al’s CORA exploratory study [19] compared their intervention development process (a posteriori) with recommendations highlighted by Darlow and Wen’s review [23], which recommends the adoption of 8 practices in the development of mobile phone interventions. In addition to the steps described above, user testing was conducted via qualitative methods, adequate time needed to test technology was anticipated, stakeholders were engaged in all steps of the intervention design, usability of the app to ensure the technology was simple and intuitive was assessed, the intervention’s promotion of a sense of competence over patients’ own care was explored, health professionals were consulted to ensure the use of the mobile app was not a burden to them, and the results of development and testing phase were published.

Schofield and Chambers’s framework [24] specifies 7 features for the development of effective, clinically feasible, and sustainable interventions: (1) targeting cancer type and stage, (2) tailoring to unique individual needs, (3) promoting self-management, (4) efficient intervention delivery, (5) ensuring evidence-based and theoretical grounding, (6) specifying protocol training and adherence, and (7) confirming stakeholder acceptability. All the previously described steps were followed in the design of Pereira-Salgado et al’s REMIND system study [20].

The importance of using a theoretical grounding in the design of mobile phone interventions was highlighted by this review. Murray et al’s conceptual model [25], which provides a description of multidimensional factors affecting medication adherence, was used to inform the strategies used by the CORA mobile app [19]. Self-determination theory informed the use of motivational interviewing as part of the nurse phone support strategy in Pereira-Salgado et al’s REMIND system study [20]. Self-determination is a theory of motivation that emphasizes the importance of supporting individuals’ natural tendencies to exhibit healthy behaviors [26].

Social cognitive theory [27], more specifically self-efficacy, guided the content design of the SMS messages in Spoelstra et al’s intervention [17]. According to the authors, messages were written using motivational content to stimulate the participants’ engagement with SMS and behavior change. In Pereira-Salgado et al’s REMIND system study [20], motivational interviewing provided as part of the nurse support was designed to stimulate participants’ self-assessment of the problem as well as to help provide them with the information, resources, and skills needed to achieve oral chemotherapy adherence. The authors indicated that the nurse phone support strategy appeared to increase self-efficacy according to the analysis of participants’ interviews, but this was only assessed qualitatively.

### Utility of Mobile Phone Interventions in Increasing Adherence to Oral Chemotherapy

Due to research designs employed by the reviewed studies and the aims of this scoping review, numerical comparisons are not offered. This section provides a narrative approach to describe findings on the observed utility of mobile phone interventions in improving adherence to oral chemotherapy.

The 2 experimental studies in this review (Spoelstra et al’s study and Greer’s et al’s CORA experimental study) [17,18] did not find statistically significant differences between the experimental and control groups. However, findings point toward patient and treatment variables (high levels of anxiety, poor baseline adherence, and length of treatment), which may moderate the effect of interventions on oral chemotherapy adherence. Participants who reported adherence problems at baseline showed better adherence after using the app than the standard care group (as measured by Medication Event Monitoring System) [18]. This study also found that participants with high levels of anxiety in the experimental group showed better adherence to oral chemotherapy than the standard care group at the end of the study (measured by Morisky Medication Adherence Scale). Spoelstra et al’s study [17] found that participants in the experimental group showed better adherence than the control group in later weeks of the study (measured by SMS reply self-report).

Pereira-Salgado et al’s REMIND system study described participants’ perceived utility of the intervention in increasing adherence to tyrosine kinase inhibitors [20]. Most participants reported that reception and response to SMS reminders stimulated their medication adherence due to accountability (eg, reinforcing habits at the beginning of treatment or drug intake support during time of routine change).

Although qualitative studies [19-21] were not designed to evaluate the effect of interventions on medication adherence, they constitute a necessary step in the development of acceptable, usable, and relevant interventions, which were also found useful to participants in supporting their oral chemotherapy intake.

### Issues and Limitations Related to the Use of Mobile Technology

Failure to receive up to 40% of SMS on time was experienced by 2 out of the 9 participants who completed Pereira-Salgado et al’s study because of slow networks in rural areas [20]. Technological difficulties and being without their mobile phone (eg, left at home and losing phone) were reported by some participants. These barriers seem difficult to overcome and should be taken into consideration at the time of designing interventions using mobile phones.

One limitation found in the use of the CORA mobile app [19] was the need to send symptom reports to clinicians via email instead of using the electronic health record system due to regulations. This method did not guarantee that clinicians would open the report emails when sent. Another limitation of the app was the support of only iPhone and Android phones, excluding other operating systems. The authors recognized the potential to include other smartphone operating systems to reach a broader population of smartphone users.

Brett et al’s study did not describe limitations and issues related to the use of their mobile app [21]. This study was a conference proceeding, which can explain reduced information about the topic.

## Discussion

### Principal Findings

This scoping review brings together the available evidence on adherence-enhancing interventions delivered via mobile phone in the context of oral chemotherapy. A total of 5 studies describing 4 interventions met the inclusion criteria. This low figure may be because the widespread use of oral chemotherapy is a relatively new medical advancement, and the extended access to mobile phones, especially smartphones, is also a recent phenomenon, which can also explain the young data of studies in this area.

Consistent with trends in other chronic diseases [6], this review shows that the 2 main features used to deliver mobile phone interventions aiming to increase oral chemotherapy adherence are SMS and mobile apps. Regardless of the technology feature employed, all interventions explored were highly acceptable, useful, and feasible to be implemented in clinical settings.

Despite the variety of adherence-enhancing strategies in the interventions, 2 strategies were common to most studies: drug-related symptom management advice and reporting and medication-intake reminders. This approach is compatible with evidence on drug-related symptoms and forgetfulness as the 2 main barriers to oral chemotherapy adherence [33]. It is important to notice that although all the reviewed interventions primarily addressed adherence barriers related to the patient (forgetfulness, knowledge of therapy, and condition) and the therapy (side effects), only half of those interventions took into account health care team and system-related barriers (communication with treating team, monitoring of adherence, and side effects). Strategies to address these barriers consisted of reports on the presence and severity of side effects and adherence frequently sent to treating teams to stimulate prompt communication and adequate monitoring of oral chemotherapy treatment as required [18,20].

The relevance of patients’ involvement in the design, implementation, and evaluation of health research has been widely recognized [34]. The use of a participatory research model allows generation of more significant research questions, alignment of intervention goals with end users’ needs, increase in the acceptability and usability of health interventions, and enhancement of translation of findings into real-life settings. This was acknowledged by most studies in this review whose intervention strategies were shaped by stakeholders’ perceptions of barriers to adherence, need for self-management support, or their preferences on components of the interventions. It was also generally recognized that interventions need to be found to be acceptable, useful, and usable to stakeholders before moving toward experimental research phases.

This review showed that there were no established processes for the development of mobile phone health interventions. Some researchers did not use or at least did not report the utilization of a mobile phone intervention design framework, including theoretical grounding. Following a framework to design mobile phone–based adherence-enhancing interventions in the oral chemotherapy context supports the development of acceptable interventions that are of intuitive and relevant use to cancer patients. Overall, the use of design frameworks can help to adequately plan the resources needed in each stage of the design as well as to canalize these assets into tools that can be successfully implemented in oncology settings.

Mobile phone health intervention design frameworks in this review also highlight the need to develop interventions based on a theoretical approach. This is crucial as technology alone cannot be seen as a strategy to increase medication adherence. Although most reviewed studies reported a theoretical framework informing their design, some inconsistencies were found in the explanation of the theoretical elements of the interventions, for example, the use of the term “motivation” alone to describe self-efficacy-informed SMS content or intervention strategies. According to Bandura’s self-efficacy theory [27], individuals’ levels of motivation are heavily based on their beliefs in their capacity to display behaviors that will impact events affecting their lives. Therefore, motivation alone may not be enough to explain the influence of self-efficacy-based interventions on medication adherence. Moreover, in the context of self-efficacy theory, patients’ success in adhering to oral chemotherapy is the most effective source informing patients of their ability to follow their drug prescriptions. It is crucial for self-efficacy-based interventions to describe the process through which self-efficacy (as a construct influenced by multiple elements) is expected to support cancer patients to achieve adherence to oral chemotherapy. Without this explanation, motivation remains an isolated variable that cannot be linked to self-efficacy.

Overall, general perceptions of mobile phone technologies in this review were positive. As an example, SMS had high rates of delivery and response success, presenting this mobile phone feature as one that is able to be successfully implemented in clinical settings. However, the use of SMS and other mobile phone features encompass challenges that are not easy to overcome. Patients who live remotely, with poor internet or phone coverage, are prone to miss medication reminders or experience issues accessing mobile apps. Patients may also lose their mobile phones or leave them at home, missing the opportunity to benefit from real-time interventions at times. At the time of reporting results related to adherence-enhancing interventions delivered via mobile phones, it is important for authors to describe strengths, limitations, and barriers in the use of mobile phone technology. This will help to inform future researchers on the obstacles and advantages of delivery features when designing interventions of this type.

A strength of this review is its novelty as it is the first study to examine the current state of knowledge about oral chemotherapy adherence–enhancing interventions delivered via mobile phones and to identify opportunities for future research in the area. Another strength of this study is the use of a methodological framework for scoping reviews, which increases consistency and structure of the search process and reporting of findings. In addition, reliability of the search strategy was increased by involving a research librarian in the process.

### Limitations

Scoping reviews include a variety of study types to answer broad research questions by mapping available evidence and identifying knowledge gaps. The purpose of scoping reviews is not to ask highly focused research questions or to assess the quality of the reviewed literature, as is the case in systematic reviews. Due to the variety of research designs in the reviewed studies, quantitative analyses on available data were not possible.

The search strategy in this study was limited to research published in English, which may have led to the omission of other sources of information.

Furthermore, this review was able to incorporate and analyze only those studies available at the time of the search that fit the inclusion criteria. Our search yielded results showing 1 study protocol that despite meeting most of the inclusion criteria, was not research-based at the protocol stage [35].

### Opportunities for Future Research

This review provides evidence of the scarcity on studies that evaluate the effect of mobile phone interventions on adherence to oral chemotherapy. Mobile phone interventions in this review were highly acceptable and useful to oral chemotherapy users. Therefore, there is a need for future research to take the next steps into experimental studies to generate evidence-based knowledge that has the potential to be translated into oncology settings.

In other chronic diseases, the use of SMS medication reminders has proven to be effective in increasing medication adherence [28]. It would be useful if future studies carefully described the key elements of SMS reminders used in interventions (eg, content, frequency, and sender) so that researchers are able to determine which elements are most likely to have an impact.

It is possible for SMS reminder interventions to incorporate content grounded on evidence-based theoretical models that encourage behavior change. In addition, due to internet accessibility, use of smartphones enables text message interventions to deliver not only medication reminders but also larger contents of information addressing additional barriers to oral chemotherapy (eg, education).

Despite a general failure of studies to report cost-effectiveness analysis of mobile phone adherence tools [6], the design of these types of interventions may involve elevated costs in time, human, and financial resources. Due to this consideration and the need for adherence-enhancing tools to be translatable to real oncology settings, future research could benefit from following a mobile phone health intervention design framework and the inclusion of economic analysis.

According to the World Health Organization, patients in developing countries face a number of health care barriers (eg, short staffed hospitals, lack of patient access to care, and long waiting times to see a doctor) [29], which may increase the chances of oral chemotherapy nonadherence. It is estimated that in developing countries in 2015, one-third of people owned a smartphone, and this figure is set to increase to approach the ownership rates in developed countries in the next few years [30]. Studies in this review focus exclusively on patients living in developed countries. It would also be useful to explore the impact of such interventions in developing countries.

Although the scope of this review was not limited to adults, the body of literature included in this study only targeted cancer patients older than 18 years. Evidence shows that adolescents and young adults are at higher risk of oral chemotherapy nonadherence than younger and older users [31]. The use of mobile phones among adolescents and young adults is even higher than that among adults. In Australia, in 2015, 9 in 10 teenagers (aged 14-17 years) owned a mobile phone [32]. Therefore, it is important that future research also addresses the nonadherence of younger oral chemotherapy users via mobile phone–delivered interventions.

More studies on the moderator effect of anxiety, poor baseline adherence, and length of treatment would be beneficial to understand the role those variables play on oral chemotherapy adherence interventions delivered via mobile phone.

### Conclusions

This review shows the lack of research on oral chemotherapy adherence–enhancing interventions delivered via mobile phone. Available interventions, delivered via SMS and mobile apps, are highly acceptable and useful to oral chemotherapy users, and nonadherence in this group is a serious issue. These findings support the need for the development and evaluation of mobile phone tools to assist cancer patients to follow their oral chemotherapy prescriptions. This review also highlighted the importance of stakeholders’ involvement and the use of a design framework in the development of mobile phone–based interventions aiming to support oral chemotherapy intake to increase translatability into real oncology practices. Given the increasing use of oral chemotherapy and the widespread availability of mobile phones worldwide, further research in this field is expected to rapidly increase in the near future.

## Appendix

Multimedia Appendix 1 Included studies.

## Abbreviations

CORA: ChemOtheRapy Assistant
RCT: randomized controlled trial
SMS: short message service
