# Supplementary material for: Mobile-Based Oral Chemotherapy Adherence–Enhancing Interventions: Scoping Review
Source: JMIR Mhealth Uhealth. 2018 Dec 21;6(12):e11724. doi: 10.2196/11724 (PMC6320412; doi:10.2196/11724)
Supplement: Multimedia Appendix 1 [file mhealth_v6i12e11724_app1.pdf]

n	Author/ year	Study purpose	Research Design	Participants	Mobile Phone Technology	Intervention	Main Findings
1	Spoelstra et al. 2016	To examine proof of concept of a text messages intervention and to conduct a preliminary evaluation of the efficacy of text messages with respect to adherence and symptom severity and interference in adult cancer patients prescribed oral anti-cancer agents.	Randomized controlled trial (2:1 allocation ratio), longitudinal (10 weeks).	75 participants newly prescribed oral chemotherapy or oral hormonal therapy with diverse types of cancers. Most common were: breast, prostate, lung, colon, and multiple myeloma. Recruited at two community cancer centres and a large specialty pharmacy in the United States of America.	Text message reminders.	21 days of short message service for adherence, control group received care as usual.	Differences in adherence between the experimental and control group were not statistically significant. Acceptability: 57% (83 of 145) of eligible participants consented to take part in the study, 88% (37/42 participants) in the experimental group read the text messages most of the time and 90% (38/42 participants) were satisfied with the intervention.<br><br>Limitations: Challenges in the use of self-report tools to measure medication adherence as this relies on participants ability to recall medication intake.
2	Greer et al. 2017	To test the effect of a smartphone mobile app to improve adherence and symptom management in patients prescribed oral chemotherapy.	Randomized controlled trial with two groups, longitudinal (12 weeks).	181 patients with diverse cancers prescribed oral chemotherapy.<br><br>United States of America.	A mobile phone app (designed for the study).	Intervention group: 12 weeks of using mobile app and usual care. Control group: usual care.	Study groups did not differ across outcome measures from baseline to week 12.<br><br>Higher adherence scores Medication Event Monitoring System (MEMS) were observed in patients who reported adherence problems at baseline in experimental group compared to control group.<br><br>Patients with higher anxiety assigned to the

							experimental group reported better adherence on the 12 week Morisky Medication Adherence Scale (MAAS) compared to standard care.
3	Fishbein et al. 2017	To develop and evaluate the usability and acceptability of a smartphone app to support adherence to oral chemotherapy and symptom management in patients with cancer.	Qualitative	32 stakeholders (cancer patients /caregivers, oncology clinicians, cancer practice administrators, representatives of community)<br>10 patients using oral chemotherapy (app's alfa testing).<br>5 patients using oral chemotherapy (app's beta testing).<br><br>All in the United States of America.	Mobile phone app (designed for the study)	Focus groups with 32 stakeholders to inform the design of the app.<br>Individual semi-structured interviews with 10 oral chemotherapy users and 8 oncology clinicians (alpha testing of app).<br>Individual semi-structured interviews with 5 oral chemotherapy users (beta testing of app).	Stakeholders and patients who participated in alfa and beta trials of the app informed the inclusion of the following components in the app:<br>medication reminders, self-reporting of medication adherence and symptoms, an education library including nutritional information, Fitbit integration, social networking resources, and individually tailored symptom management feedback.<br>Usability and acceptability feedback during alfa and beta testing also informed the development of the app.<br><br>Limitations:<br>Quantitative usage and acceptability data during the development and preliminary testing phase, was not collected.<br><br>The mobile app was only available for iPhone and Android operating systems, with potential to be modified in the

							future to include other operative systems.
4	Pereira-Salgado et al. 2017	To develop and pilot-test the clinical feasibility and acceptability of a mobile health system (REMIND) to increase oral drug adherence and patient symptom self-management among people with chronic myeloid leukaemia (chronic phase).	Qualitative, longitudinal (10 weeks)	10 patients with chronic myeloid leukaemia (chronic phase) and 2 nurses recruited from a cancer centre in Australia.	Text message reminders and web app.	Participants used the REMIND system for 10 weeks. The REMIND system consisted of daily text message reminders to take medication, weekly side-effect assessment online with automatically generated self-care advice and phone-based nurse consultation sessions.	Most patients indicated that receiving and responding to the text reminders prompted medication adherence due to accountability.<br><br>The usability of the intervention was high. Most participants expressed ease with text message reminders and symptom report.<br>Benefits reported by patients: help in establishing medication routines, resolution of symptom uncertainty, increased awareness of self-care, and informed decision making.<br>Nurses also endorsed the intervention: it assisted in establishing pill-taking routines and patients developing effective solutions to adherence challenges.<br><br>Limitations and recommendations:<br>Failure to receive up to 40% text message reminders was reported by 2/9 patients who completed the pilot study. This was due to slow networks especially in remote areas.<br>Data saturation might not have

							been reached due to sample size. This study included only patients diagnosed with chronic myeloid leukaemia. Future studies need to pilot this intervention in other cancer diagnosis.
5	Brett et al. 2018	To explore the feasibility of an app to improve adherence in women prescribed adjuvant endocrine therapy following breast cancer.	Qualitative (1 month)	20 women prescribed adjuvant endocrine therapy following breast cancer, recruited through the UK charity Breast Cancer Care and Independent Cancer Patients' Voice in the United Kingdom.	Mobile app (designed for study)	1 month trial of the app.	The app was found easy to download and use.<br><br>Participants found the information section useful but expressed their willingness to receive more information on side-effect management.<br><br>Links to evidence-based information and adjuvant endocrine therapy were perceived by participants as reassuring.<br><br>Participants stated that recording their side effects in the app's diary helped them to manage their symptoms.<br><br>Positive feedback was provided about medication reminders.<br><br>Perceptions of the usefulness of a chat forum were mixed.

References:

1) Spoelstra SL, Given CW, Sikorskii A, et al. Proof of concept of a mobile health short message service text message intervention that promotes adherence to oral anticancer agent medications: a randomized controlled trial. Telemed J E Health. 2016 Jun; 22(6):497-506 PMID:26716365

2) Greer J, Jacobs JM, Pensak N, Nisotel LE, Fishbein J, MacDonald J, Ream M, Fuh C, Buzaglo JS, Muzikansky A, Lennes IT, Safren S, Pirl WF, Temel JS. Randomized trial of a smartphone mobile app for adherence to oral chemotherapy. JCO 2017 May 20; 35(15_suppl):10055-10055
3) Fishbein JN, Nisotel LE, MacDonald JJ, Amoyal Pensak N, Jacobs JM, Flanagan C, Jethwani K, Greer JA. Mobile application to promote adherence to oral chemotherapy and symptom management: a protocol for design and development. JMIR Res Protoc 2017; 6(4): e62. PMID: 28428158
4) Pereira-Salgado A, Westwood JA, Russell L, Ugalde A, Ortlepp B, Seymour JF, Butow P, Cavedon L, Ong K, Aranda S, Breen S,Kirsa S, Dunlevie A, Schofield P. Mobile health intervention to increase oral cancer therapy adherence in patients with chronic myeloid leukemia (The REMIND System): clinical feasibility and acceptability assessment. JMIR Mhealth Uhealth 2017 Dec; 5(12):e184. PMID: 29212628
5) Brett J, Watson E, Boulton M, Stoner N. Exploring the potential for a smart phone app to improve adherence to adjuvant endocrine therapy (AET) in women after treatment for breast cancer. Psycho-Oncology 2018; 27 (S2):20-21. DOI:10.1002/pon.4639
